# Handling in the Production of Wire-Based Linked Micro Parts †

**DOI:** 10.3390/mi8060169

**Published:** 2017-05-25

**Authors:** Philipp Wilhelmi, Christian Schenck, Bernd Kuhfuss

**Affiliations:** BIME—Bremen Institute for Mechanical Engineering, MAPEX Center for Materials and Processing, University of Bremen, Badgasteiner Str. 1, 28359 Bremen, Germany; schenck@bime.de (C.S.); kuhfuss@bime.de (B.K.)

**Keywords:** multi-stage micro part production, linked parts, diameter adaptive guides

## Abstract

For simplified processing and the enhancement of output rate in multi-stage production, micro parts are handled as linked parts. This contribution discusses handling specific challenges in production based on an exemplary process chain. The examined linked parts consist of spherical elements linked by wire material. Hence, the diameter varies between the wire and part. Nevertheless, the linked parts must be handled accurately. The feed system is an important component too, but special focus is given to the guides in this present study. They must adapt to the diameters of both the parts and the linking wires. Two alternative variants of adaptive guides are presented and investigated under the aspects of precise radial guiding, vibration isolation, damping behavior and friction force.

## 1. Introduction

**Figure 1:**
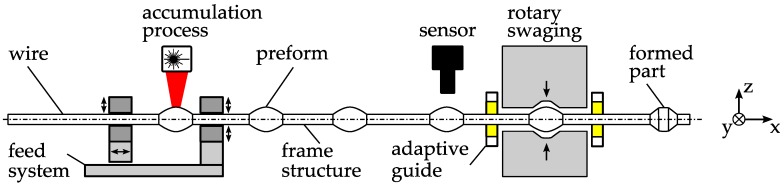
Example of wire-based linked micro part production.

Handling constitutes a major challenge in the production of micro parts with regard to achieving an economic and efficient production. Due to the typically low price per unit, high outputs and consequently low cycle times are required. At the same time, precise positioning is necessary as the parts are very sensitive to mechanical damage and size effects [1]. For example, dominating adhesive forces may complicate handling. Van Brussel et al. [2] provided a broad overview of the assembly of microsystems, in which handling is a main aspect. Adhesive forces are primarily derived from electrostatic attraction, Van der Waals forces, and surface tension. These forces depend on environmental conditions and can also be used for pick-up operations, but unfortunately complicate handling. Adhesion results in inaccurate grasping and negatively affects releasing. Grasping devices in general have been addressed in a previous study [3]. Tichem et al. [4] gave a good overview of the micro-grip principles, while Fantoni et al. reviewed releasing strategies [5]. These challenges led to the development of new micro manufacturing and assembly concepts with adopted and integrated machine designs [6,7]. A complete micro bulk forming system was presented and analyzed by Arentoft et al. [8]. They considered not only the forming system itself but also all process steps, including handling. Subsequently, in another study [9] examining this bulk forming system, a new handling system that maximizes the clock rate to up to 250 strokes per minute was presented and characterized. Furthermore, Fleischer et al. [10] identified the supply of parts as a major bottleneck in micro assembly and presented a modular vibratory conveyor system, including options for separation, quality control, discard lock out and part orientation. A measure to facilitate handling and enable a production at higher output rates is the production in linked parts, which was introduced in a previous study [11]. This is especially important for micro forming processes and their special requirements [12,13], where multiple different operations may be performed on a single part. Applications of this approach to bulk forming based on sheet metal have been presented previously in the literature [14,15]. In contrast, the work presented in this paper addresses bulk forming based on wire material. Figure 1 shows an example of the production of wire-based linked micro parts. In the first process, preforms are generated within a wire by a laser material accumulation process [16]. The wire is melted partially while being pushed together. The remaining sections of the wire build the frame structure that links up the preforms. In a second step, the preforms are formed by micro rotary swaging [17,18].

To enable the production of linked parts at high output rates, appropriate production facilities are developed and investigated. The feed system is essential for the accumulation process. It must provide a high dynamic positioning with adequate precision and accurate alignment of the wire within the process zone. The chosen solution in this case is a gripper-based feed system driven by a linear direct drive. The grippers are self-centering and aligned to each other by the design concept. The gripper geometry provides good support of the wire by line contact. Nevertheless, the distance of the preforms within the linked parts may vary due to changes in length of the frame structure during a multi-stage production process, especially when forming is included [18]. Hence, a referencing of the individual parts becomes necessary for the positioning in a subsequent production process. Therefore, a visual high-speed part referencing system has been developed [19]. The system is based on a line camera, which is triggered based on the signal of the position sensor of the feed system. Appropriate guides are important to guide the linked parts securely through the process chain, and such guides are also addressed in this paper.

## 2. Requirements for Adaptive Guides

**Figure 2:**
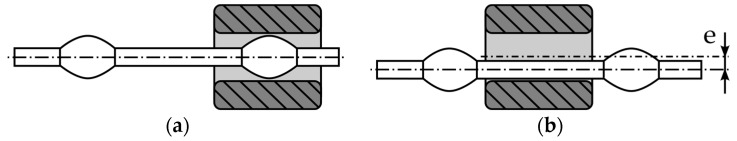
Misalignment in the case of applying a classical wire guide bushing: (**a**) part within the bushing; (**b**) only wire within the bushing.

Depending on the processes and their spatial alignment, the guides along the process chain must meet different requirements. It is important that the guides suppress vibrations that occur between these processes. The part must be centered within the tools, close to the process. The vibrations induced by the process should be damped and the transmission of vibrations from the process to the linked parts outside the tool and vice versa must be suppressed. Furthermore, a compact size can be important due to space limitations. Conventional wire guides are typically realized as bushings consisting of ceramic materials. They are especially optimized for high wear resistance. As they are implemented as sliding guides, they usually feature a clearance. The clearance cannot be downscaled linearly to the micro range, which is related to tolerancing issues in micro production, as discussed in a previous study [20]. This problem already exists in the case of guiding raw wire material, because the diameter tolerances are too high. However, this becomes even more evident when considering the standard deviation of the preform diameters, which depend on the actual process parameters. Furthermore, with a constant bushing diameter, the clearance depends on the position of the parts, which can lead to a significant misalignment of the center line of the linked part (Figure 2). The linked parts used for the presented investigations feature a frame diameter of *d*_frame_ = 360 µm and a part diameter of *d*_part_ = 660 µm. Hence, assuming that there is no clearance between the guide and parts, the maximum possible misalignment is *e*_r_ = 150 µm for this example.

The measure to overcome this problem by increasing the length of the guide beyond the maximum distance between the parts is limited by the following reasons. First, due to space limitation, this solution is not always applicable. Furthermore, the nearest guiding point in relation to the tool would depend on the position of parts in this case. Considering the low structural stiffness of the linked parts, precise guiding cannot be guaranteed this way. Adaptive guides are investigated to solve the described problems, which can locally adapt their diameter to the actual needs [21]. The required features of such a guide can be summarized as follows: precise radial guiding with sufficient stiffness, good re-centering ability after displacement of the linked parts, high damping of radial and axial vibrations, low and preferably constant resistant forces against feed direction, compactness, high wear resistance, ability of lubricant free operation, and reasonable manufacturing costs by good manufacturability. Generally, the design of micro bearings is strongly influenced by the manufacturability. Nevertheless, studies on air bearings [22,23], magnetic bearings [24,25], and ball bearings [26] can be found. All of these guiding solutions have two well-defined partners with constant geometrical properties. In the field of high accuracy requirements in combination with geometrical adaption, only gripper-based concepts are mentioned. A simpler solution with limited travel is the application of flexure hinges, which further offer the advantage of zero clearance and therefore are also applied for micromanipulation devices [27].

## 3. Concepts of Adaptive Guides

**Figure 3:**
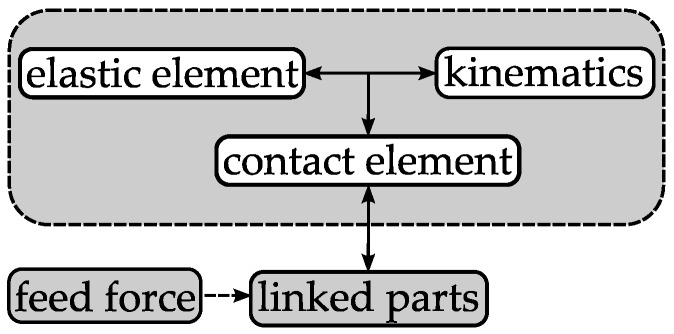
Passive adaptive guide function scheme.

The general function of a linear guide is to limit the original six degrees of freedom of the guided part. In this present study, this means a translation in the feed direction and a rotation around the center line. By this definition, a wide solution space is spanned. Adaptive bearings can be either active or passive [28]. An active adaptive guide controls the clearance by measuring it with a sensor and adapting the diameter with an actuator comparable to the aforementioned grasping solutions. Regarding the needed components, such a system is comparatively complex. The complexity obviously becomes a major challenge when considering the huge number of adaptive guides that are required for the production of linked parts. Hence, a passive solution is more suitable and the presented concepts in this paper are limited to pure mechanical systems. A function scheme of a mechanical passive adaptive guide is illustrated in Figure 3. It uses the feeding force to increase the guide diameter when a part passes through. It consists of three elementary functional elements: a contact element allows relative motion and guarantees wear resistance; a kinematics transforms the feeding force to a radial force, which acts on an elastic element to enable the diameter adaption. In the macro range for high precision applications, a high stiffness is usually preferred. In the considered case, a reduced stiffness has less impact on the accuracy, due to the inherent low rigidity of the linked parts.

**Figure 4:**
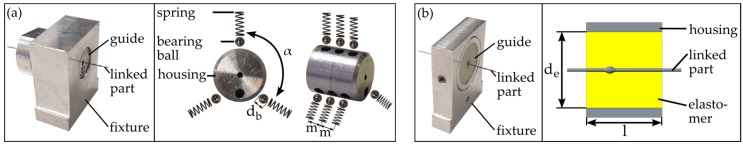
(**a**) Concept G1, *d*_b_ = 2 mm, α = 120°, *m* = 3 mm, spring rate *R* = 2.122 mN/mm; (**b**) Concept G2, *d*_e_ = 16 mm, *l* = 8 mm, material: Polyurethan, 70 Shore A.

Based on this scheme, two prototypes were built. Concept 1 (see Figure 4a) uses bearing balls and spiral springs. The kinematics is realized by the radius of the bearing ball and the contour of the parts. The advantage of this solution is that the components are available as standard parts with high quality. Based on the characteristics of the used components and the considered linked parts, the nominal preload of the guide is *F*_frame_ = 0.34 N for a frame diameter of *d*_frame_ = 360 µm. The force of one spring on a part of diameter *d*_part_ = 660 µm is *F*_part_ = 0.66 N.

The maximum simplification with respect to the number of components is realized by concept 2 (see Figure 4b). A cylindrical elastomer element with a central bore integrates all functions in one component. In order to realize the preload, the bore diameter is chosen to be slightly smaller than the frame diameter, *d*_frame_. The advantage of the simplification of this concept goes along with the disadvantage of the coupling guiding features, such as stiffness and wear resistance. They depend on the applied material. In the following, the concepts are called G1 and G2.

## 4. Measurements and Results

The requirements formulated in the previous section can be divided into two groups. Some properties of the guides, such as compactness, ability of lubricant-free operation, manufacturability, and partial wear resistance, are more or less given by the construction. Others have a direct influence on the processes and need to be determined experimentally in detail. These aspects, which are analyzed in the following, are guiding precision, influence on vibrations, and influence on the feed force.

### 4.1. Guiding Precision

**Figure 5:**
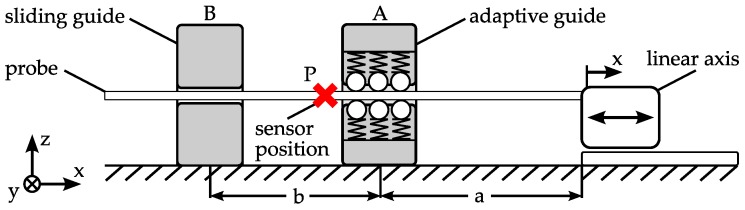
Measurement setup—radial deviation, *a* = 142 mm, *b* = 60 mm.

For the evaluation of the guiding precision, the displacement in the *z*-direction is measured while a probe is moved through the adaptive guide (Figure 5). This is conducted in two measurement series with two different kinds of probes. First, raw wire is used, and second, the same measurements are performed with linked parts. The setup consists of a linear axis mounted on a base plate with different mounting positions for the guides.

**Figure 6:**
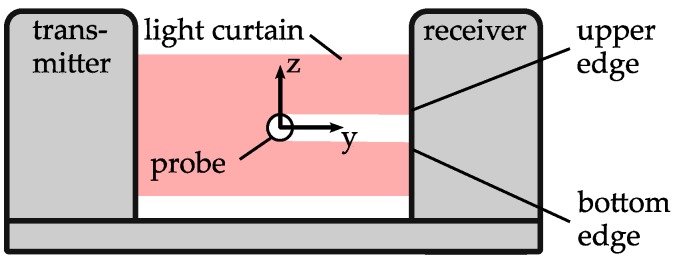
Measurement setup—radial deviation.

For each of the tests, one of the two adaptive guides, G1 and G2, as well as an additional sliding guide are used. Their positions are named A and B. The distances between A and the home position *x* = 0 mm of the linear axis as well as the distance between A and B are described by the variables *a* and *b*, respectively. The different adaptive guides are mounted in position A. Furthermore, an aluminium block with a bore of a diameter of *d*_sb_ = 400 µm is used for sliding the bushing at position B. The sensor is located at position P, directly outside the adaptive guide. The position of the probe in the *z*-direction is measured by an optical micrometer (Figure 6). The accuracy of the used device is specified to 0.7 µm. Between the single measurements, the probe is moved in the *x*-direction by the linear axis. The displacement between two measurements determines the measurement point distance Δ*x* relative to the wire. The sensor detects the upper edge of the probe. Consequently, the measurements consider only the *z*-direction. Assuming that the used probe is not rotationally symmetric, a different behavior in the *y*- and *z*-directions is expected and the angular orientation of the probe around its length axis (*x*-axis) could influence the measurement results. Consequently, two measurements are performed for each test configuration. First, a measurement with the same probe section and probe orientation is performed for both guides. Following this, the probe is rotated 90° around its length axis (*x*-axis) and the measurements are repeated. For each measurement, the probe is fed in fixed steps of distance Δ*x* until reaching position *x* = 10 mm and measured for each step, before being pushed back to the starting position. This measurement procedure is repeated 10 times. The radial deviation e is calculated by relating each measurement to the initial value at the position of *x* = 0 mm. The centering force of the guides is assumed to be direction-independent. Hence, the orientation of the guides is considered negligible. Nevertheless, G1 is oriented during testing in a way so that the uppermost of the three springs is aligned with the *z*-axis.

**Figure 7:**
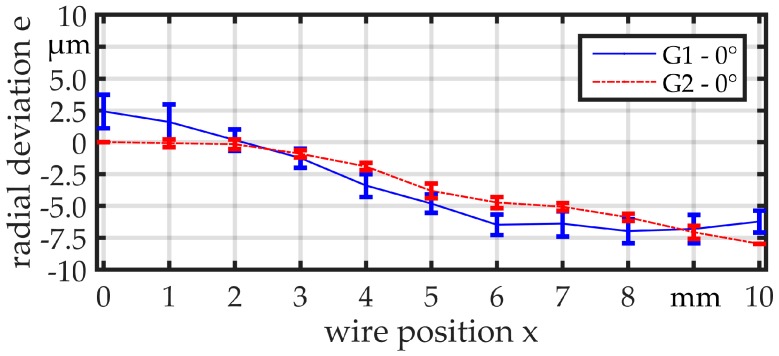
Comparison of radial deviation for G1 and G2 measured with wire, *d*_wire_ = 360 µm.

**Figure 8:**
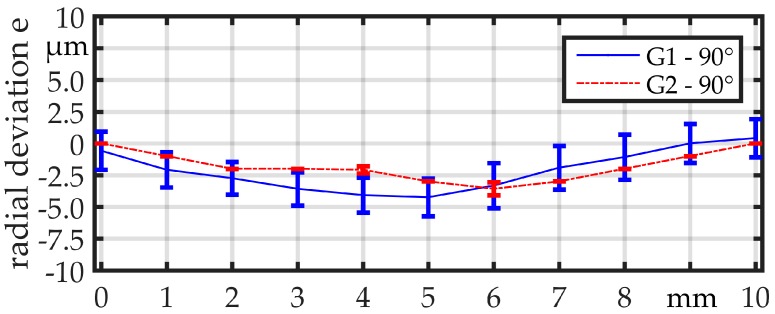
Comparison of radial deviation for G1 and G2 measured with wire, *d*_wire_ = 360 µm.

For a proper interpretation of the measurements, it is important to distinguish different influences. One influence is expected to be the contour accuracy of the probe, which is not necessarily rotationally symmetric. Therefore, the base material of the linked parts, which is hard-drawn wire (1.4301/AISI 304) with a diameter of *d*_wire_ = 360 µm with bright surface produced in bars, is used instead of the linked parts for the first experiments. The measurement point distance is set to Δ*x* = 1 mm. Figure 7 and Figure 8 illustrate the results. The range of the mean value from the minimum to the maximum is beneath 10 µm for the orientation of 0° and beneath 5 µm for the orientation of 90° for both guides. The standard deviation of the measurements is close to the sensor resolution, but obviously smaller for guide G2. Within the diagrams, the measurements show a similar curve shape for the same angular orientation but different guides. Comparing the curve shapes between the two diagrams respectively, a clear difference can be observed between the different angular orientations. This can be explained by the fact that even the raw wires show some contour deviations due to being not ideally straight and rotationally symmetric.

**Figure 9:**
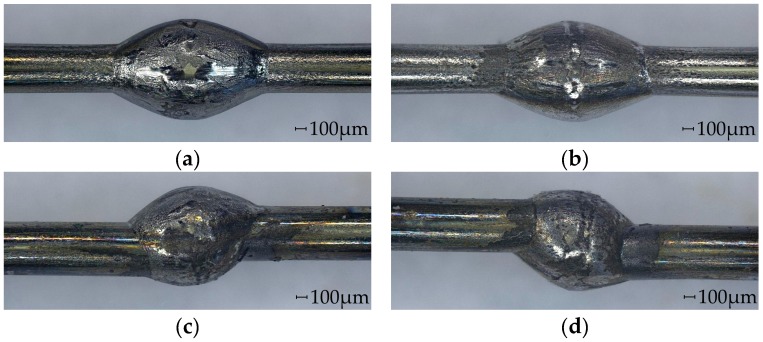
(**a**) Linked part 1 at 0°, (**b**) Linked part 1 at 90°, (**c**) Linked part 2 at 0°, and (**d**) Linked part 2 at 90°.

Finally, G1 and G2 are tested with real linked parts. For further identification of the influence of the contour of the parts, two different linked parts are used (Figure 9). Linked part 1 shows a good rotational symmetry and no measurable offset *o*_p1_ ≈ 0 µm between the two wires at both sides of the part. Linked part 2, in contrast, shows an offset *o*_p2_. The frame diameter of *d*_frame_ = 360 µm and the part diameter of *d*_part_ = 660 µm is equal in both cases. The length of the parts is *l*_part 1_ ≈ 1200 µm and *l*_part 2_ ≈ 1000 µm. The measurement procedure is the same as before, but the measuring point distance is reduced to Δ*x* = 0.2 mm. Furthermore, the measurement is again performed two times for each linked part and, for the second measurement, the linked part is rotated 90° around its length axis (*x*-axis). The parts are illustrated in Figure 9 in both orientations. The measured offset of linked part 2 is *o*_p2,0°_ = 165 µm for the orientation of 0° and *o*_p2,90°_ = 225 µm for the orientation of 90°.

**Figure 10:**
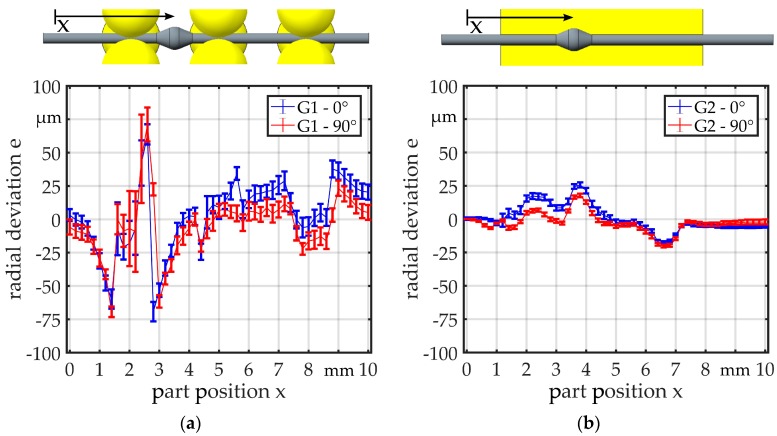
Radial deviation for (**a**) G1 and (**b**) G2 measured with linked part 1 (no offset between wire ends).

**Figure 11:**
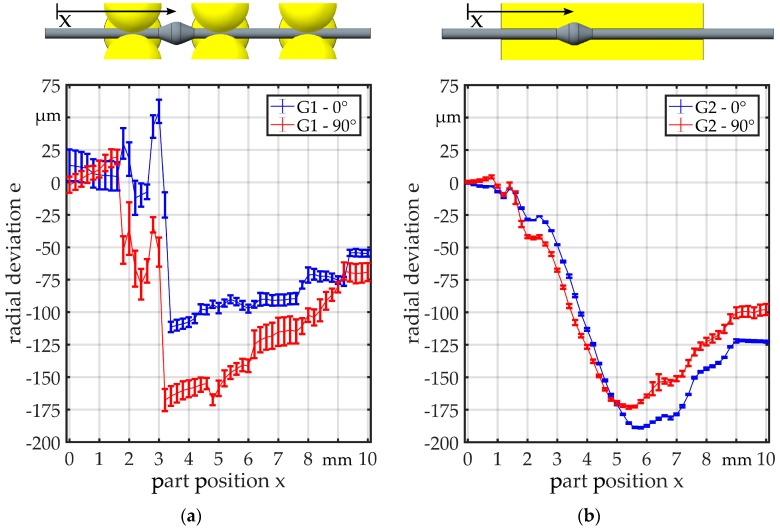
Radial deviation for (**a**) G1 and (**b**) G2 measured with linked part 2 (offset between wire ends).

In the measurements with G1 (Figure 10a), the regions where the bearing balls are in contact with the parts can clearly be recognized by the unsteady curve. The range of the deviation is about 40 µm in the zones where only the wire is in contact with the bearing balls. The overall range of the deviation is about 160 µm. A similar behavior can be observed in the measurements for G2 (Figure 10b), but the curve is smoother. The overall range of the deviation is about 50 µm. Before the part enters the guide (*x* = 1 mm) and after it has passed through (*x* = 9 mm), the deviation e is close to 0 µm. The curves for the two different angular orientations are quite similar. As expected, the deviation for linked part 2 is much higher and in a range of 225 µm (Figure 11). The deviation is on a constant level, when the part is not within the guide, but there is a significant offset between the two described regions when the part is before and when it is behind the guide. For both guides, the curves are similar, but for G2, the curve is smoother and shows a smaller standard deviation.

### 4.2. Vibrations

**Figure 12:**
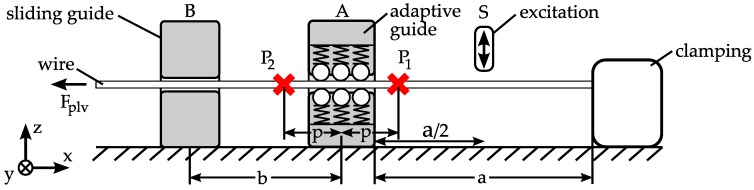
Setup vibration analysis, *a* = 230 mm, *b* = 60 mm, *p* = 17 mm.

For the vibration analysis, an application-specific testing is performed, which allows a qualitative comparison of the different guide concepts under the aspect of suppression of radial vibrations (Figure 12).

**Figure 13:**
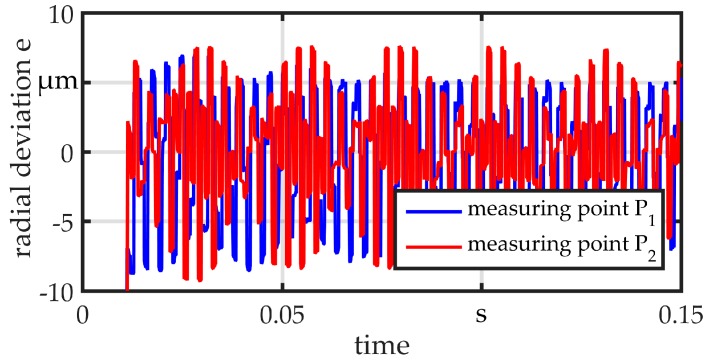
Vibration measurement sliding guide *d*_sg_ = 0.8 mm.

**Figure 14:**
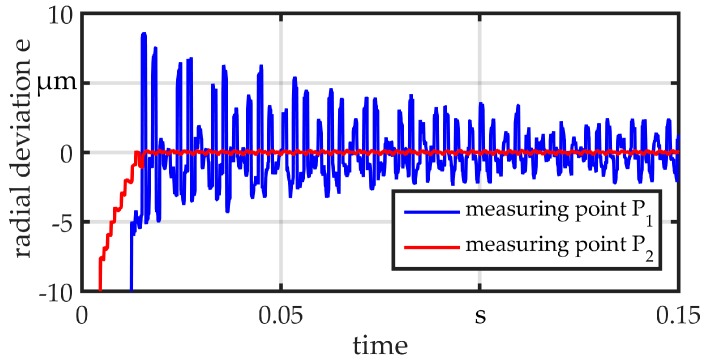
Vibration measurement guide G1.

**Figure 15:**
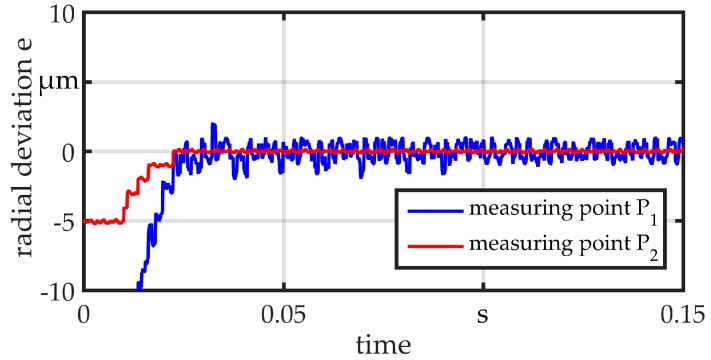
Vibration measurement guide G2.

All measurements are performed with a raw wire with a diameter of *d*_wire_ = 360 µm. The wire is clamped at one end, fitted within a sliding guide (position B) and preloaded with *F*_plv_ = 25 N at the other end. The different guides are mounted in position A during testing. At point S, the wire is excited by displacing and releasing it in a repeatable way, so that it vibrates in its eigenfrequencies. The wire is simultaneously measured at the points P_1_ and P_2_. The same sensor in previous measurements is used and the initial value is set to zero in order to calculate the radial deviation e. For comparison, a standard non-adaptive sliding bushing with a diameter of *d*_sg_ = 800 µm is used. Results are shown in Figure 13. The bushing provides a clearance of 0.22 mm between the wire and guide, as may be necessary in the worst case for a linked part. The wire can almost vibrate freely on both sides of the guide. The amplitude is about 8 µm. The results for the guides G1 and G2 are illustrated in Figure 14 and Figure 15, respectively. Both show a good suppression of radial vibration for the wire segment behind the guide. Considering the vibrations at point P_1_, G2 shows a better damping, as could be expected for an elastomer. In the measurements, no vibration can be seen. There is only a variation in the range of the sensor resolution.

### 4.3. Feed Force

**Figure 16:**
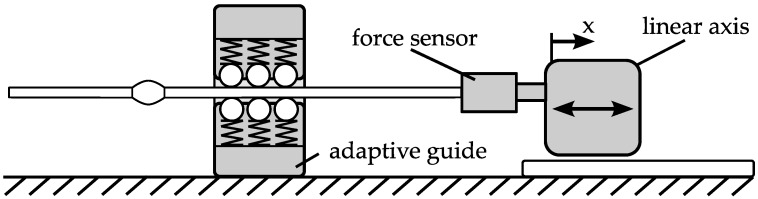
Measurement setup—feed force.

The developed passive principle is based on the idea of using the feed force for a diameter adaption of the guide. When this adaption is realized by spring elements with a constant spring rate, the feed force changes during feeding. Measurements are performed with a force sensor (Figure 16). The feed distance is 20 mm, so that the part is able to completely pass through the guide.

**Figure 17:**
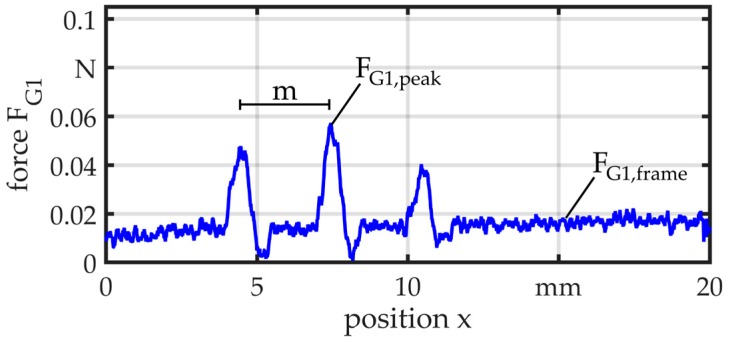
Force measurement—G1.

**Figure 18:**
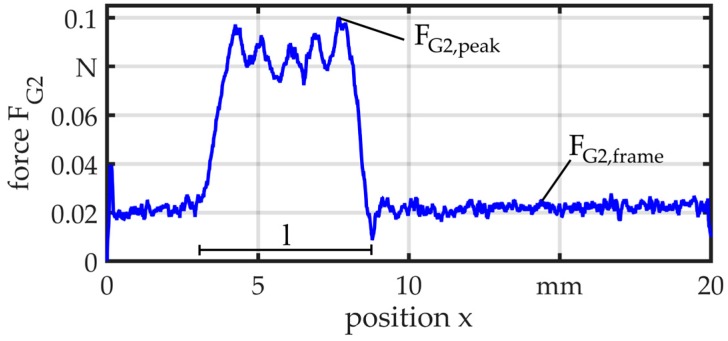
Force measurement—G2.

Figure 17 and Figure 18 illustrate two exemplary results at a feed velocity of *v* = 10 mm/s. In both measurements, the wire is tensioned at the start of the motion and the force rises quickly. Only the frame moves through the guide and the force is relatively constant with *F*_G1,frame_ = 0.02 N and *F*_G2,frame_ = 0.03 N. In the measurements of G1, the force peaks of *F*_G1,peak_ = 0.06 N appear when the part comes in contact with the bearing balls. The distance *m* ≈ 3 mm between the balls can be recognized in the measurement. For G2, the force rises up to *F*_G2,peak_ = 0.1 N when the part passes through the guide. In this case, there is a little difference between the zone of elevated force and the length of the guide, which is *l* = 8 mm. This can be explained by the lower axial stiffness of the elastomer element.

## 5. Conclusions

A concept for the passive and adaptive guiding of linked parts has been developed. Based on the concept, two prototypes were built and examined under the aspects of guiding precision, suppression, and damping of radial vibrations as well as influence on the feed force.

It is shown that adaptive guides improve the guiding precision compared to non-adaptive guides, but the precision is still influenced by the contour of the parts that are guided. This influence is more or less distinct depending on the guiding concept. Regarding the feed force, an influence of the adaption is a direct consequence of the passive nature of the guiding. The feed force is used for the radial adaption and, consequently, the adaptive guides cause force peaks when parts pass through. These peaks can be clearly observed. They are low in comparison to the forces that are delivered by typically applied feed devices. Nevertheless, a reduction by further optimization of the kinematics is aspired to decrease the amplitude of these peaks. Otherwise, these force peaks could induce vibrations to the system and decrease the accuracy. In the vibration measurements, it is shown that both guide concepts are generally suited to suppress the transmission of vibrations between different sections of the linked parts. Concerning the damping of vibrations close to a process guide, G2 shows a better performance. Based on the developed function scheme, further concepts can be realized to further improve the damping behavior.

## Supplementary Materials

The following are available online at www.mdpi.com/2072-666X/8/6/169/s1.
